# Feasibility of a new homebased ballistocardiographic tool for sleep-assessment in a real-life context among workers

**DOI:** 10.3233/WOR-211312

**Published:** 2023-04-18

**Authors:** Jennie Nyman, Elin Ekbladh, Mathilda Björk, Peter Johansson, Jan Sandqvist

**Affiliations:** a Department of Health, Medicine and Caring Sciences, Linköping University, Norrköping, Sweden; b School of Wellbeing, Metropolia University of Applied Science, Helsinki, Finland; c Pain and Rehabilitation Center, Department of Health, Medicine and Caring Sciences, Linköping University, Linkoping, Sweden

**Keywords:** Objective assessment, occupational health, vocational rehabilitation

## Abstract

**BACKGROUND:**

There is a need for simple and suitable tools for assessing sleep in a natural home environment.

**OBJECTIVES:**

This study explores the feasibility in terms of implementation and acceptability of a new homebased ballistocardiographic (BCG) tool for objective sleep-assessment in a real-life context.

**METHODS:**

The participants included thirty-nine workers, taking part in two seven nights’ sleep-assessment periods. Objective data regarding sleep was collected with BCG. Subjective data regarding sleep was collected with a sleep diary. Implementation was analysed by determining the number of nights with usable signal quality and comparing with the total number of potential nights and by exploring associations between objective and subjective sleep data. Acceptability was analysed by categorizing the participants’ experiences of how the BCG tool impacted the sleep.

**RESULTS:**

In terms of implementation, usable BCG data increased from 40% at assessment phase 1 to 70% during assessment phase 2. Moreover, in assessment phase 2, there was a significant moderate correlation between the ‘time in bed’ assessed by the BCG and in sleep diary by participants in the first five nights. In terms of acceptability, almost one third of the participants did not experience any impact of the BCG on the sleep. Two participants experienced a major negative impact on the sleep.

**CONCLUSIONS:**

This study indicates that the novel BCG tool could be feasible for objective assessing of sleep in workers natural home-environment in the future, but there is still a need for development of the BCG both regarding technology and implementation process.

## 1 Introduction

Early detection of sleep disorders and interventions could improve self-rated health and work performance [1]. Thus, there is a need for suitable tools for assessing sleep and early detect sleep and stress related problems as a part of the occupational health services.

Disturbed sleep and sleep disorders have a great effect on people’s lives and perceived life balance. Problems with sleep, or sleep and wake disorders such as insomnia, may lead to functional and occupational difficulties (e.g. risk factor for sick leave and work accidents) [2, 3] and severe health conditions [2]. The results of a study by Åkerstedt et al. [4] suggest that long-term exposure to work stress results in an increased level of sleep disturbances. Perceived stress related to work has increased during the last decades and stress, regardless of the cause, leads to major costs for employers [5]. Both quantity and quality of sleep are commonly affected by stress. In a concept analysis on rest and restorative sleep [6] it is discussed that a good balance between quality and quantity of rest and sleep might give the best result and brings forward that non-restorative sleep, or sleep of poor quality, can be detrimental to health in many ways.

Sleep can be assessed both subjectively and objectively and is most commonly assessed subjectively by using tools such as sleep diary, sleep logs and sleep questionnaires [3, 7] and these types of methods are also used in occupational health [8]. The use of subjective information only when assessing sleep has however been criticized, since it contains risks for subjective bias [3, 7].

Objective measurement of sleep can be performed in different ways. Polysomnography is the gold standard for assessment of sleep and is normally performed in a sleep laboratory [7]. Polysomnography monitors several different body functions during sleep and provides detailed information about the sleep period and the sleep structure, but the method is expensive and complicated [7, 9]. An actigraph is a simpler tool, typically worn on the wrist or ankle, that can be used to assess sleep objectively. This device can be used in a home-environment over longer periods of time but can mainly be used to estimate sleep-wake patterns [10]. Neither of the tools is good enough, since one is too complicated and expensive to be used at home over longer periods of time, and the other does not give all the information needed.

However, there is now also a new novel tool, ballistocardiography (BCG), that has shown potential in performing sleep assessment in the natural home environment [11–14]. This tool is a sensitive accelerometer, measuring the movements generated by the body’s recoil from cardiac activity, and estimating sleep parameters similar to polysomnography [12, 13]. An advantage is that this tool does not require contact with the person’s body and it is affordable and lightweight [13]. Thus, BCG may be a useful tool for occupational health professionals in order to perform objective assessments of sleep in the natural sleep environment of employees. Before implementing this type of device in occupational health practice, there is a need to evaluate them. However, to the best of our knowledge only small-scale technological studies have been carried out for assessment of sleep by using BCG and no scientific studies have tested or evaluated BCG as a tool to assess sleep from an occupational health perspective previously.

In situations when there are none or only few studies using a specific intervention, such as the use of BCG among workers, performance of feasibility studies is important. Feasibility studies are carried out in order to ensure the acceptability and practicality of study implementation and to reduce threats to the validity of outcomes, before carrying out largescale studies [15, 16]. Ideally, feasibility studies are performed in a real-life context [15].

The aim of this study was to explore the feasibility of a new homebased BCG tool for sleep assessment in a real-life context, among a group of workers. Feasibility was explored, as described by Bowen et al. [15], from the perspective of implementation, i.e. the extent and likelihood that the intervention can be implemented as planned, and from the perspective of acceptability, i.e. how the intended individual recipients react to the intervention.

The research questions in regards to implementation were: 1) To what extent is the BCG tool able to generate usable data in the real-life context? and 2) Is there a relationship between objective and subjective data regarding time in bed and total sleep time? The research question in regards to acceptability was: 3) What are the experiences of the participants about how the assessment affected their sleep?

## 2 Methods

This study was part of a larger project named ‘Occupational well-being at all ages’. The overall aim of the project was to explore new novel ways of supporting people at work in order to increase occupational well-being and prevent sick leaves.

### 2.1 Design

For this study, we used a descriptive observational design with two measure points, referred to as assessment phase 1 and assessment phase 2, together with a convenient sample.

### 2.2 Ethical considerations

The study was approved by the ethical committee of Pirkanmaa Hospital District in Finland (nr R1706). All participants took part on a voluntary basis and provided written informed consent.

### 2.3 Participants

All currently working blue- and white-collar workers in a middle-sized Finnish enterprise were invited to take part in this study. An e-mail with information on the study was sent out by the human resources department to all the employees that currently were not on fulltime sick-leave or parental leave (N = 72). The workers were invited to a group meeting, held by the principal investigator (JN) and a technician specialist, where they got detailed information about the study and the research process. Everyone interested was included in the study and signed an informed consent. The total number of participants included were 39, with an average age of 44 (range 24–61), of which 27 (69%) were female and 34 (87%) white collar workers.

### 2.4 Data collection

**Fig. 1:**
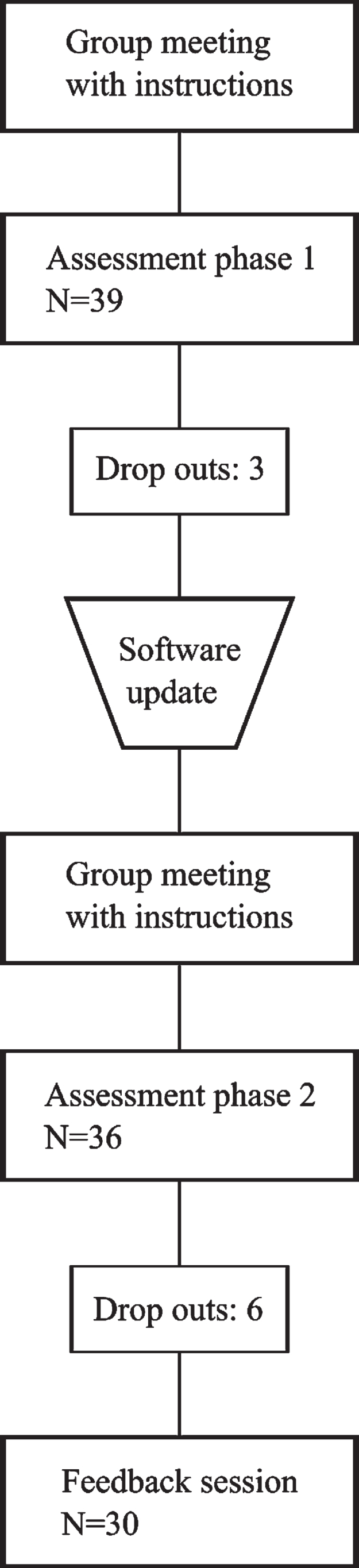
Process flow chart.

The data collection consisted of two parts carried out in October 2017 and in January 2018. The assessment phase 1 was a one-week (7 nights) sleep assessment period with both objective and subjective data collection. Based on analysis of phase 1, the BCG tool was updated and instructions clarified for assessment phase 2. The assessment phase 2 consisted of a one-week (7 nights) sleep assessment period with both objective and subjective data collection, together with a 20-minute feedback session. The assessment phase could start on different weekdays for the participants. The data collection process is presented in Fig. 1.

### 2.5 Collection of objective sleep data

**Fig. 2:**
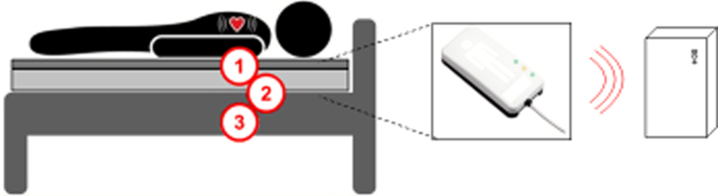
Murata SCA11H BCG equipment. Numbers 1, 2 and 3 indicate where the sensor could be placed.

Sleep was objectively assessed by using a ballistocardiographic (BCG) tool (Murata SCA11H) which is a sensitive accelerometer, or a sensor, attached to the bed frame or mattress without any direct contact to the person measured (Fig. 2). Through a software (Murata sleep and recovery analysis), a summary of total sleep, deep sleep, REM (rapid eye movement) sleep and time in bed is generated for every night together with a number showing total recovery.

**Fig. 3:**
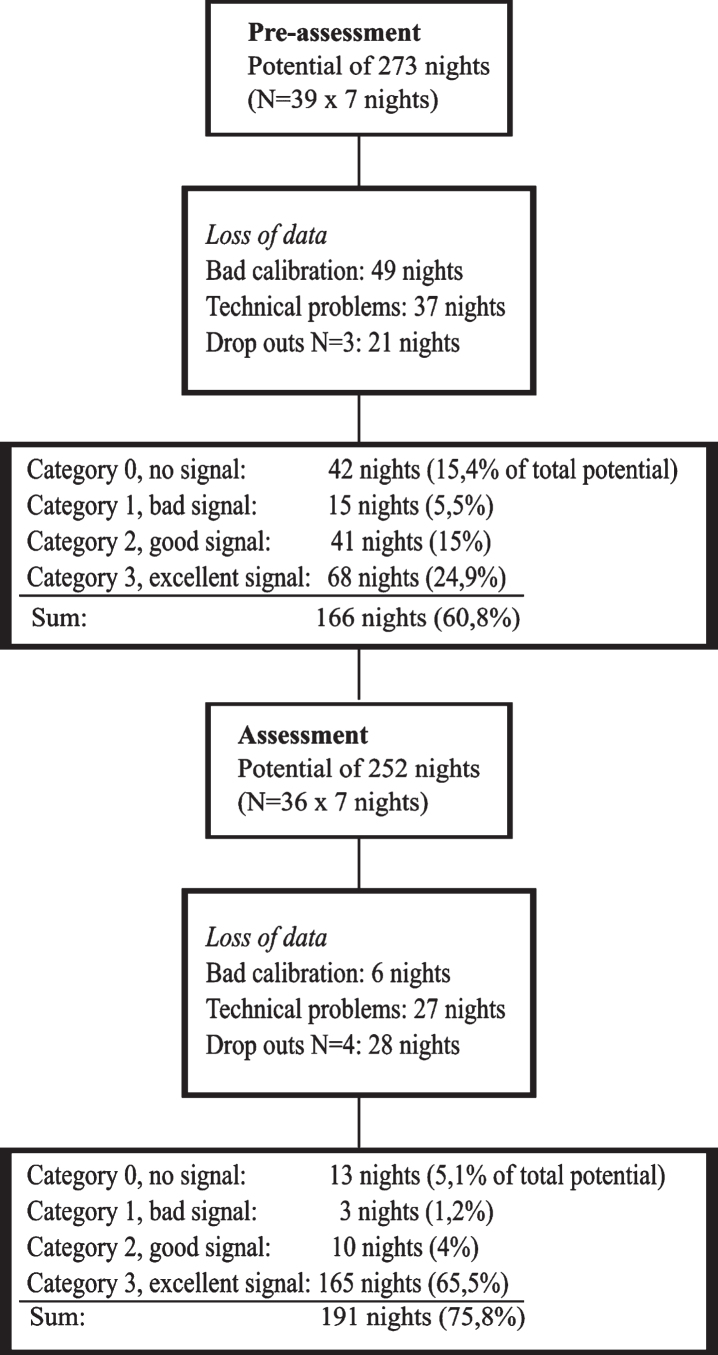
Results presented in a flow chart.

The individual participants were given a BCG sensor, together with a linked data logging device (Raspberry Pi 3 model B) and instructions how to install and calibrate the equipment, during a group meeting. The BCG sensor and data logging device automatically connected to each other via Wi-Fi, but required individual calibration in the specific sleep environment and the participants were provided with detailed instructions for this.

All devices were checked in laboratory conditions and the set up and calibration process was pre-tested with three persons, not included in this study, in advance in order to ensure optimal functioning.

Firstly, the participant had to carry out an ‘empty bed -calibration’, which means turning on the devices and staying still (not touching the bed or making any other movements), for one minute. Secondly, the participant had to lay down on the bed and start the one minute ‘in bed -calibration’, where the sensor recognizes the person in the bed. The data logging device has a light indicating which phase is ongoing or if there is a disconnection or something else needing attention. After the initial calibration the equipment did not require any attention unless the light indicated differently (for example a red light indicating a disconnection, requiring re-calibration). The participants were instructed to write down the time of calibration (-s) and they were also given the opportunity to get assistance over the phone if needed.

After each assessment phase, the equipment was returned to the researcher. The sensor data was processed by a technician specialist connected to the research group, using the sleep and recovery analysis software.

If the assessment phase 1 was successful, a new calibration was only needed if participants changed bed from assessment phase 1 to phase 2.

### 2.6 Collection of subjective sleep data

Subjective data regarding sleep was collected parallel to objective data collection, in a sleep diary developed and used in research by the Finnish Occupational Health Institute [8]. The diary consists of two questions regarding falling asleep and maintaining sleep during the night: “How long did it take you to fall asleep?”, “For how long were you awake during the night in total, from the time you fell asleep until you got up?”, and two questions regarding the sleep period: “At what time did you go to bed?”, “At what time did you get up?” Participants filled out the diary every morning. The answers generated numerical data regarding the time spent in bed and the perceived total sleep time of each night.

Approximately one month after the sleep assessment period of phase 2, the participants took part in a feedback session where the sleep assessment was discussed. During the session, the participants were asked to describe if and how the equipment or performed sleep assessment in any way affected them or their sleep. The feedback session was led and documented by the principal investigator (JN).

## 3 Analysis

The quantitative data was analyzed using the IBM SPSS Statistics 25. The extent to which the BCG tool was able to generate usable data was analyzed by determining the number of nights with acceptable signal quality and comparing with the total number of potential nights. In the summary generated by the sleep and recovery analysis software for each night, the signal quality during the sleep period was determined. According to the manufacturer, the signal can be considered usable when it reaches a designated level at least 50% of the time. The signal quality was established by categorizing into four different groups as follows: 0 = no signal; meaning that the signal was at the designated level less than 30% of the time, 1 = bad signal; at the designated level 30–49% of the time, 2 = moderate signal; 50–79% of the time and 3 = good signal; 80–100% of the time. Categories 2 and 3 were considered usable data, and therefore used in the analysis. The number of nights with usable data quality were compared between assessment phase 1 and assessment phase 2.

In order to analyze the relationship between objective and subjective data, non-parametric methods were used. Mean and inner quartiles were used in order to describe ‘time in bed’ and ‘total sleep’ for each night. Spearman’s rho was used to test the correlation between ‘time in bed’ and ‘total sleep’ assessed by BCG and by participants in the diary. We categorized the strength of correlations according to Landis & Koch’s classification [17]: 1.00–0.81 = almost perfect, 0.80–0.61 = substantial, 0.60–0.41 = moderate, 0.40–0.21 = fair and 0.20–0.00 = poor. For this analysis we only used data from categories 2 and 3, i.e. data with substantial or moderate signal strength, collected during assessment phase 2.

The participants’ experiences of how the BCG tool affected their sleep was analyzed through categorizing of the subjective data generated from the feedback session. Data was categorized based on the level of impact into four groups: ‘no impact’ = the participant did not experience any impact at all, ‘minor impact’ = the participant experienced no impact after making some adjustments, ‘moderate impact’ = the participant felt that the sleep was disturbed for a few nights, and ‘major impact’ = the participant felt that the sleep was disturbed for the whole period.

## 4 Results

### 4.1 Implementation in terms of amount of usable data

The total amount of usable data was 40% during assessment phase 1 and increased to 70% during assessment phase 2. Data collection that was successful for all seven nights of individual participants increased from 15 cases during assessment phase 1 to 23 cases during assessment phase 2. Category 3 or good data, increased with 41% of total number of potential nights, from 68 nights (of a potential of 273) during assessment phase 1 to 165 nights (of a potential of 252) during assessment phase 2. Details regarding these results are presented in Table 1.

**Table 1:** Results of amount of usable data generated in real life context

**Assessment phase 1**
Number of potential nights (N = 39×7 nights): 273
*Loss of data*
Bad calibration: 49 nights
Technical problems: 37 nights
Participant drop outs N = 3:21 nights
Number of potential nights after loss of data: 166 (60,8 % of total potential)
Category 0, no signal: 42 nights (15,4% of total potential)
Category 1, bad signal: 15 nights (5,5%)
Category 2, moderate signal: 41 nights (15%)
Category 3, good signal: 68 nights (24,9%)
**Assessment phase 2**
Number of potential nights (N = 36×7 nights): 252
*Loss of data*
Bad calibration: 6 nights
Technical problems: 27 nights
Participant drop outs N = 4:28 nights
Number of potential nights after loss of data: 191(75,8 % of total potential)
Category 0, no signal: 13 nights (5,1% of total potential)
Category 1, bad signal: 3 nights (1,2%)
Category 2, moderate signal: 10 nights (4%)
Category 3, good signal: 165 nights (65,5%)

Assessment phase 1 consisted of several unsuccessful calibrations and there were also technical problems in some of the BCG tools that did not appear during the control in lab-conditions before using in real life context, due to which data was lost. As a result from the findings from the assessment phase 1, the software of the BCG tool was updated and all devices were tested in order to ensure optimal functioning during assessment phase 2. For assessment phase 2, the participants were also advised to use tape or a similar adhesive to fix the device to the mattress if it seemed to move around too much.

### 4.2 Implementation in terms of associations between objective and subjective data

The results are presented separately for each night in Table 2. Of the seven nights recorded during the assessment, there was a moderate to substantial correlation between the ‘time in bed’ assessed objectively by the BCG and subjectively by participants in the first five nights.

**Table 2:** Correlation between objectively measured (BCG) and subjectively measured (diary) sleep data during assessment 2. Data presented separately for each night

Night	BCG time in bed (hh:mm) md(iqr)	Diary time in bed (hh:mm) md(iqr)	*r*	*p*	BCG total sleep (hh:mm) md(iqr)	Diary total sleep (hh:mm) md(iqr)	*r*	*p*
1 *n* = 27	8:33 (7:52–9:39)	8:00 (7:20–8:30)	0.518^**^	0.006	6:29 (5:30–7:42)	7:20 (6:35–8:10)	–0.064	0.751
2 *n* = 26	9:30 (8:43–10:34)	8:00 (7:37–8:41)	0.524^**^	0.006	7:09 (6:09–8:50)	7:40 (6:45–8:18)	–0.143	0.485
3 *n* = 25	9:36 (8:25–10:46)	8:20 (7:25–9:00)	0.533^**^	0.006	7:18 (6:20–9:00)	7:35 (6:45–8:37)	0.211	0.312
4 *n* = 25	10:06 (9:17–10:51)	8:50 (7:52–9:05)	0.686^**^	0.000	7:47 (5:43–9:34)	8:15 (7:35–8:45)	0.400^*^	0.048
5 *n* = 25	9:36 (8:38–11:03)	8:35 (8:00–10:05)	0.446^*^	0.029	7:08 (5:33–8:41)	7:35 (7:06–8:42)	–0.075	0.727
6 *n* = 23	9:21 (8:16–11:42)	7:50 (7:10–9:00)	0.288	0.182	7:43 (5:31–9:35)	7:30 (6:40–8:25)	0.018	0.934
7 *n* = 24	8:54 (8:08–10:20)	7:50 (7:16–8:13)	0.324	0.122	6:58 (5:14–8:31)	7:30 (6:50–7:53)	–0.096	0.655

In the analysis of the association between ‘total sleep’ as measured by BCG and reported in the sleep diary, a fair correlation was found in one of the seven nights. Night four showed strongest correlation with a substantial correlation for ‘time in bed’ and a fair correlation for ‘total sleep’.

### 4.3 Acceptability in terms of the participants’ experiences on sleep impact

Of the 36 participants assessed, 30 chose to participate in the feedback session and the experiences from these participants were categorized. Of these, nine were categorized into the ‘no impact’ group, but one of the nine brought forward that their spouse was disturbed by the sleep assessment. Eight participants were categorized into the group ‘minor impact’. They needed to make some adjustments to the equipment in order to not be disturbed by it (e.g. placing the device differently, using tape in order to keep the device in place). Nine of the participants were categorized into the group ‘moderate impact’. Specific for this group was that the participants felt disturbed for a few nights, especially during the first data collection period. They did not sleep as well as normally, as the sleep was more restless and/or the assessment came into mind during the night. Some also thought about the diary and the fact that they would have to remember how many times they woke up at night or for how long they were awake. This made the participants pay attention to these factors at night, which then affected their sleep negatively. There were three participants experiencing a ‘major impact’. Two of these clearly stated that they were affected negatively throughout both assessment phases, as they could not get the sleep assessment off their minds and their sleep was more restless than normally. The third participant in this category experienced a positive effect and brought forward that sleep was better than normally during both sleep assessment periods. One participant did not fit into any of these categories. This participant had major problems with the set-up of the equipment and that became the main experience.

## 5 Discussion

In this study, we used the perspective of Bowen et al. [15] when exploring the feasibility of a new homebased BCG tool for sleep assessment in a real-life context. The feasibility was explored from the perspectives of implementation and acceptability. We found that that this could be an acceptable tool for assessing sleep in a home-environment but both the technology and the process needs further development before it could be implemented in practice.

Focusing on implementation in feasibility studies means, according to Bowen et al. [15], studying the extent, manner and likelihood that an intervention can be implemented as planned. Successful installation and calibration are crucial for a successful assessment, and in this study these factors were explored through the extent to which the BCG tool was able to generate usable data. The results of this study show that the installation and calibration were successful in 70% of the cases. The risks of an unsuccessful installation need to be minimized, or even eliminated, if this tool is to be used in a similar way with a larger, and possibly more diverse group of people. One way of enhancing the calibration process is to use an internet-based solution for data transfer and storage, like in the study by Sadek and Mohktari [13]. An internet-based version gives the professional the opportunity to follow the installation and calibration process in real time and guide the participant through possible problems right away, which could result in more data on good quality sleep in the end. However, an internet-based version requires certain standards of the equipment (e.g. smart phone and a good internet connection), which we were not able to provide for all participants within this study. In order to be able to provide all participants with equal possibility to take part in the study, we had to make choices, like using the more affordable local mini-computer for data transfer and storage, which gave us less possibilities to control the calibration process.

Improvements of the BCG with regard to technology and use of the tool are needed before implementation in clinical practice can be suggested. Though we were able to increase the amount of usable data from assessment phase 1 to assessment phase 2 fairly well, we still missed 30% of the registrations. As technology develops, devices such as BCG might become more complex on the inside. However, it is important that this development does not interfere with the usability for the users [18].

Another aspect of the implementation that we looked into was, if the tool could provide correct data when used in a home-environment. We therefore explored correlations between subjective and objective data for ‘time in bed’ and ‘total sleep’, using only good quality data collected during the assessment phase 2. For the most part, there was a significant moderate correlation between the ‘time in bed’ measured by BCG and the ‘time in bed’ reported by the participant. The ‘time in bed’ was a concrete variable and therefore suitable to look at in this feasibility study. The last two nights showed only fair correlations. The reason for this could be that the participants over time might get less precise when filling out the diaries.

There were significantly more differences in ‘total sleep’. Only one night had a significant fair correlation between BCG reported and participant reported time. ‘Total sleep’ is a more complex variable to measure both in BCG and in diary format than ‘time in bed’. Guidelines for evaluating and managing insomnia bring forward that symptoms, for example time awake during the night, may be difficult to estimate correctly by the person [3]. In addition, the questions in the diary regarding the time spent awake during the night seemed unclear for some participants, and may withhold a bias due to that. In order to get a coherent picture of sleep, objective measuring is important [3]. Therefore ‘total sleep’ should be estimated further, for example by comparing BCG assessments with polysomnography which is the gold standard.

According to the theoretical perspective of Bowen et al., acceptability refers to how the intended individual recipients, both participants and others involved, react to the intervention [15]. In this study, we carried out assessment of sleep by using the BCG tool in the homes of a group of working people. In the assessment of sleep, it is of importance to not disturb the participants’ sleep. This may be achieved by the use of simple tools such as the BCG in the participants’ home. However, in our study 12 participants out of 30, i.e. 40%, were clearly affected by the sleep assessment, either moderately or majorly. Similarly, in a previous study [13] BCG technology is presented as “non-intrusive” but when looking into the results of that study, one of the three participants was afraid of the new system at first. This indicates a need to go beyond the physical dimensions of sleep assessment in order to really understand the extent to which the BCG-method is affecting the individual. In order for this tool to be acceptable and affecting the sleep as little as possible, it seems important to give the person enough information and time in order to get used to it before starting the actual assessment.

Perceived stress at work has increased during the last decades [5] and it has been suggested that working under stress for a long period of time leads to a risk of bad sleep quality and high fatigue [19]. Prolonged sleep disturbances, such as insomnia, may lead to severe health conditions of both somatic and mental character [2, 20]. Subjective assessment of sleep is not enough [3, 7] and therefore occupational health professionals need objective tools also. BCG tools could be a possibility for several reasons. Firstly, they estimate sleep parameters similar to polysomnography, which is the gold standard. Secondly, they could be easy to use in a home-environment which is where people sleep normally. And thirdly, they are not attached to the body which gives the possibility of a less disturbed sleep. Early detection of sleep problems is crucial and since sleep is easily disturbed, it is of importance to develop tools and assessment procedures that are reliable, easy to use, and interferes with the sleep as little as possible.

### 5.1 Limitations and future research

This feasibility study was carried out at an early phase of the development of the tool, with limited resources and with a small convenient sample of working people. Feasibility from the perspective of practicality [15] would be good to explore further, in order to get an understanding of actual resources needed for a large-scale study with an internet-based solution. Richer data collection on how the assessment affected the life and sleep of the participants and their family members could give a deeper understanding of what to consider when assessing sleep of the participant in a home-environment. The tool also needs to be used in studies among different client groups in order to test its feasibility more broadly, before carrying out a large-scale study.

## 6 Conclusions

This study indicates that the novel BCG could be a feasible method for assessing sleep in workers natural home-environment. On this tool, there are very few studies published and therefore there is still a need for development of the BCG both regarding technology and implementation process. Objective assessment methods suitable for use in occupational health need to be explored and developed from the perspective of usability. In the future the BCG tool shares the promise to be useful in occupational health settings for assessing service users’ sleep in an easy manner in their own natural home-environment.
